# A firearm bullet lodged into the thoracic spinal canal without vertebral bone destruction: a case report

**DOI:** 10.1186/1752-1947-5-289

**Published:** 2011-07-06

**Authors:** Jamal Hossin, Morteza Joorabian, Mohammad Pipelzadah

**Affiliations:** 1 Radiology Department, Al-Assad University Hospital, Damascus University, Damascus, Syria

## Abstract

**Introduction:**

Firearm injuries account for 13% to 17% of all spinal cord injuries, and are generally caused during warfare or assault with intent to kill. Spinal cord injuries caused by firearms are usually observed in patients aged 15 to 34 years old, and are especially common among men.

**Case presentation:**

We report the case of a 28-year-old Iraqi man who was referred to our radiology department with lower limb paraplegia secondary to a gunshot wound. We performed 64-slice computerized tomography with two-dimensional and three-dimensional reconstruction of the thoracolumbar spine. On the two-dimensional and three-dimensional reconstructed axial images of the thoracolumbar spine, an intra-canalicular bullet nucleus was found at the mid-spinal cord at the T8 level, with no evidence of vertebral bone destruction.

**Conclusions:**

To the best of our knowledge, there is only one previous report in the literature describing a case of a bullet nucleus lodged into the inferior epidural spinal canal without destruction of the vertebral bone. With the rise of violence worldwide the incidence of gunshot injuries continues to increase, and, thus, it is essential for radiologists to have a clear understanding of gunshot injuries and the findings on radiographic images.

## Introduction

Gunshot wounds to the spine are potentially devastating injuries that account for approximately 13% to 17% of all spinal cord injuries every year [1]. The thoracic spine is the most commonly affected region, but gunshot injuries involving the cervical spine are the most devastating of all injuries; such injuries result in the most severe functional impairments [2]. Spinal cord injuries inflicted by firearms usually result in complete paraplegia [3]. This neurological outcome (paraplegia) results from direct trauma brought about by compression of the spinal cord by the bullet nucleus, bone fragments, and sometimes disc particles [4].

To the best of our knowledge, there is only one previous report in the literature [5] describing a case of a foreign body lodged into the vertebral canal after a gunshot injury without any accompanying destruction of bony tissues. Our case report is the second such report. The patient in the first case reported presented without neurological deficit, but our patient presented with paraplegia.

## Case presentation

**Figure 1:**
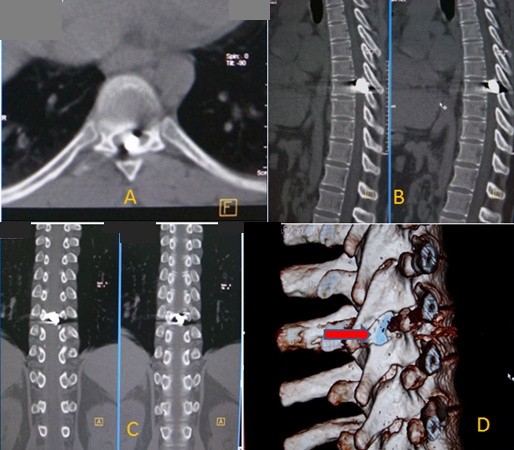
**Imaging studies of the bullet injury**. (A) Axial thoracic computed tomography (CT) image at the T8 level demonstrating the bullet nucleus in the canal without any bone destruction. Metallic artifacts can also be seen. (B) Two-dimensional sagittal reconstruction image, with the bullet nucleus in the mid spinal cord without bone destruction. Metallic artifacts can also be seen. (C) Two-dimensional coronal reconstruction CT image, with the bullet nucleus in the mid spinal cord without bone destruction. Metallic artifacts can also be seen. (D) Three-dimensional reconstruction CT image of the bullet in the intra-canalicular canal with no bone destruction. In this image the bullet setting can clearly be seen.

A 28-year-old Iraqi man was referred to our radiology department with lower limb paraplegia due to a gunshot wound. The wound occurred in the chest two days before his presentation, and was inflicted by a small-caliber handgun from a distance of approximately 6 m. Our patient was hospitalized by the emergency services after he was injured. The entrance wound caused by the traversing projectile was located at the level of the seventh rib at the left posterior axillary line. An exit wound was not observed. Neurological examination identified lower limb paraplegia. He was referred to our radiology department after initial management of the wound and after his condition became stable. We performed 64-slice computerized tomography (CT) with two-dimensional and three-dimensional reconstruction of the thoracolumbar spine. On the two-dimensional and three-dimensional reconstruction of the axial CT images of thoracolumbar spine, an intra-canalicular bullet nucleus was found at the mid-spinal cord at the T8 level, without any evidence of vertebral bone destruction (Figure 1).

## Discussion

The thoracic vertebrae have a stable biomechanical structure maintained by means of costovertebral joints and the thoracic cage. Thus, greater force is needed to cause injuries to the thoracic spine than to other regions. Because the canal of the thoracic vertebra is narrower than those of the cervical and lumbar zones, injuries to the thoracic spine may damage the spinal cord [6].

Ballistics is defined as the scientific study of projectile motion [7,8] and is divided into three categories: internal, external, and terminal ballistics. Internal ballistics is concerned with the projectile within the firearm. External ballistics is concerned with the projectile in the air. Terminal ballistics is concerned with what happens when the projectile hits its target. Wound ballistics is a subset of terminal ballistics, and is the most important aspect of ballistics that physicians need to understand [8,9].

Energy from a gunshot projectile is directly related to both the mass and the square of the velocity of the bullet (KE = 1/2 mv^2^). Gunshot energy is further classified into low and high energy, depending on muzzle velocity. Muzzle velocities of less than 1000 to 2000 feet/second (304.8 to 609.6 m/second) are defined as low energy, whereas speeds higher than 2000 to 3000 feet/second (609.6 to 914.4 m/second) are defined as high energy [2]. Low-energy firearms include pistols and handguns; high-energy and high-velocity weapons include military assault rifles [2].

It is crucial to determine the type of a weapon was used and the distance between the weapon and the victim, because treatment options will depend on these criteria. In addition to the amount of energy released from the weapon, the path of the bullet can result in severe injury because the zone of destruction may be larger than expected. Yaw refers to the tumbling of a bullet along its longitudinal axis. Therefore, long bullets produce increased yaw and can result in a large zone of destruction [10]. Our patient sustained an injury from a handgun (a low-energy firearm) from a distance of approximately 6 m.

After a gunshot injury, spinal cord defect occurs as a result of direct damage by the bullet nucleus or metallic particles, or as a result of compression by the broken bone particles. Although less commonly observed, disc material can cause neural defects by compressing the canal when the bullet nucleus damages the annulus. This results in an increase in pressure on the nucleus pulposus after the bullet nucleus settles at an inter-vertebral locus [11]. In the case of our patient, after the gunshot injury the bullet nucleus was lodged in the spinal canal in the inferior thoracic zone. There were no defects of bony tissues, but a spinal cord defect occurred owing to direct damage by the bullet nucleus. Firearm injuries in the spinal zone are generally stable [9]. If the bullet in the lumbar zone breaks the pedicle or facet while traversing, it can cause an acute or chronic instability [2]. If the pedicle or facet is intact, no spinal instability is observed. If instability is suspected, flexion and extension radiographs or CT follow-up are needed [10]. Initially, two orthogonal plain radiographic views of the spine must be obtained to locate fragments of the bullet and detect fractures. This should be followed by CT, which is optional, because it allows for more precise localization of the bullet fragments within the spinal canal or vertebral segments [11]. Use of MRI in assessing gunshot wounds to the spine is debated upon. There is legitimate concern that bullet fragments may migrate under the magnetic pull and cause additional damage and injury [12,13]. The advantages of MRI over CT include markedly less artifacts, better soft-tissue imaging, and coronal, sagittal, and axial visualization of neural elements [12,14]. In our practice, we do not routinely perform MRI unless there is clear clinical evidence of neurological deterioration. Even in such cases, a neurosurgeon should be consulted and the benefit-risk ratio should be carefully evaluated.

## Conclusions

To the best of our knowledge, there is only one previous report in the literature [5] describing a case of a bullet nucleus lodged into the inferior epidural spinal canal without any destruction of vertebral bony tissues.

With the rise of armed violence worldwide, the incidence of gunshot injuries continues to increase and, thus, it is essential for radiologists to have a clear understanding of gunshot injuries and their radiographic imaging, especially gunshot wounds to the spine that are the most problematic of all these injuries. Because of the theoretical risks associated with use of MRI and the metallic projectile, routine use of MRI for the assessment of gunshot wounds has not been advocated.

## Consent

Written informed consent was obtained from the patient for publication of this case report and any accompanying images. A copy of the written consent is available for review by the Editor-in-Chief of this journal.

## Competing interests

The authors declare that they have no competing interests.

## Authors' contributions

JH interpreted our patient's MRI results and supervised the writing of the manuscript. MJ was a major contributor to writing the manuscript. MP contributed to figure formatting. All authors read and approved the final manuscript.
